# Effects of Digital Noise Reduction Processing on Subjective and Objective (Pupillometry) Assays of Listening Effort

**DOI:** 10.3390/audiolres15050122

**Published:** 2025-09-23

**Authors:** Lipika Sarangi, Jani Johnson, Gavin M. Bidelman

**Affiliations:** 1 Department of Audiology and Speech Pathology, College of Health Professions, University of Arkansas for Medical Sciences, Little Rock, AR 72205, USA; 2 School of Communication Sciences & Disorders, University of Memphis, Memphis, TN 38152, USA; jajhns10@memphis.edu; 3 Department of Speech, Language and Hearing Sciences, Indiana University, Bloomington, IN 47408, USA; gbidel@iu.edu; 4 Program in Neuroscience, Indiana University, Bloomington, IN 47408, USA; 5 Cognitive Science Program, Indiana University, Bloomington, IN 47408, USA

**Keywords:** digital noise reduction, pupillometry, listening effort, hearing aids

## Abstract

**Background/Objectives**: Although research has demonstrated the positive impacts of hearing aid (HA) digital noise reduction (DNR), limited research is available on the impacts of the strength of DNR on listening effort. This study evaluated the effects of changes in the strength of HA DNR on listening effort, measured, behaviorally, using a self-report rating scale, and, physiologically, using pupillometry. The agreement between both measures was also examined. **Methods**: Eleven young adults with normal hearing completed a sentence-in-noise recognition task. Stimuli were processed through four noise reduction conditions (off, minimum, medium, maximum) using DNR algorithms found in conventional digital HAs. After sentence presentation, participants subjectively rated their perceived listening effort. Pupillometry was recorded during the task to assess changes in pupil size (a proxy of listening effort) during sentence recognition. **Results**: Participants’ perceived listening effort reduced as the noise reduction strength increased from off to medium DNR and then plateaued for the maximum DNR condition. Pupil dilation increased from off to medium DNR and then reduced for the maximum condition. Correlation analyses suggested no agreement between self-report and pupillometry measures of listening effort. **Conclusions**: Both self-report and pupillometry measures demonstrated changes in listening effort, with changes in the DNR strength indicating that noise reduction systems do provide benefit in reducing listening effort to a certain extent. Lack of agreement between the measures suggests that both methods might be assessing different constructs of listening effort and care should be taken while making methodological decisions to assess listening effort in individuals wearing HAs.

## 1. Introduction

Effective listening requires adequate use of peripheral hearing as well as several higher-order cognitive resources. For individuals with normal hearing, listening in quiet is a relatively effortless process. However, listening in the presence of background noise and hearing loss [1] is challenging and can result in higher cognitive load, leading to effortful listening [2]. Listening effort is defined as the deliberate allocation of mental resources to overcome obstacles to goal pursuit when carrying out a listening task [3]. In digital hearing aid (HA) users, listening effort can be alleviated to some extent by using different HA features such as digital noise reduction (DNR) algorithms [4].

Digital HAs use different technologies/algorithms to assist in improving the signal-to-noise ratio in the presence of background noise. These algorithms are designed to cater to different types of signals and listening goals. For example, adaptive directional microphones to improve speech understanding in noise, digital noise reduction to reduce steady-state noise, and other noise reduction strategies to reduce wind or transient noises. Of those, the goal of DNR is to reduce unwanted background noise to improve comfort and reduce listening effort [5]. Different HA manufacturers use different noise reduction strategies to achieve these goals. For example, some use a gain-based noise reduction and others use a more spatially-based noise reduction [6]. Although the results are mixed, there is evidence from several studies indicating the effectiveness of DNR in reducing listening effort, especially in difficult signal-to-noise ratio conditions [7]. For example, Desjardins and Doherty [7] assessed individuals’ listening effort using a sentence-in-noise recognition task as well as a self-report measure and reported that the DNR algorithm significantly reduced listening effort in more difficult listening conditions. Like individuals with hearing loss, individuals with normal hearing also benefit from DNR algorithms when listening in challenging environments. Many wearable and hearable devices other than HAs also employ similar DNR algorithms to improve listening effort [8,9,10].

Studies on individuals with normal hearing and hearing loss have used different methods to assess listening effort in response to auditory task demands. Results of these measures often show a non-monotonic association, where effort increases with increasing difficulty but then decreases once the task is too difficult or the listener loses motivation to listen [3]. These non-monotonic responses have been observed across different measures of listening effort, including self-report, behavioral, and physiological measures. Self-report measures are either in the form of questionnaires or rating forms [2,11]. They are quick, easy to administer, and can assess an individual’s perceived effort while listening to auditory stimuli [12]. Behavioral tasks are often in the form of single-, dual-, and multi-task paradigms [12]. They have the advantage of increased objectivity but can also be time-consuming and are possibly less sensitive to changes in listening effort than self-report measures [13]. Reaction time (RT) measures have also been considered as a reliable behavioral measure [14]. Reduced RTs have been reported in easier listening conditions with higher signal-to-noise ratios. This indicates that physiological measures provide an objective way of measuring listening effort. These approaches record changes in central or autonomic nervous system activity [12] such as changes in pupil dilation, heart rate and breathing, and skin sweat in response to a stressful auditory stimulus [15]. Physiological measures can either be recorded using laboratory-grade equipment [15] or wearable sensors [16] and often include more than one method to assess listening effort. However, many investigators have reported a lack of correlation between results using the different methods [17,18]. The lack of correlation might be attributed to different methods measuring different constructs of listening effort or limitations of the different methodologies altogether.

One of the most commonly used physiological methods to assess listening effort is pupillometry [19]. Pupillometry involves continuous recording of changes in pupil diameter while completing a task. As pupil size is not under the active control of the individual being tested, it reduces subjectivity bias [19] and is a popular physiological biomarker of listening effort. Pupillometry has also been established as a reliable method of measuring listening effort during an auditory task [15], where pupil dilations increase with increasing effort [20]. As pupil size reflects the contributions of the autonomic nervous system’s response to effortful tasks, it also reflects a person’s willingness to exert more effort because they believe the task is worth the effort [21]. Several studies have demonstrated that as the task demand increases, leading to more effortful listening, pupil dilation also increases [22].

Several studies have used pupillometry and simulated conditions to mimic the perceptual processing and listening effort that might occur in disordered populations and with hearing assistance technologies. For example, normal hearing young adults have been used as participants to assess the impacts of auditory spectral resolution on listening effort [23]. Results revealed increased pupil dilation with reduced spectral resolution, which is indicative of increased listening effort. Abramowitz and colleagues [24] recorded pupil dilation in young and older normal hearing individuals in response to a simulated degraded signal processed through cochlear implants and reported greater pupil dilation in older adults, indicating an impact of age. Conducting these kinds of experiments with normal hearing individuals can help better understand the effectiveness of using pupillometry and other measures to assess listening effort—as a method itself—before addressing the complexities and heterogenous consequences of hearing loss. Researchers have also used pupillometry to assess the impacts of DNR on listening effort and have demonstrated that, when DNR was activated, pupillometry showed significantly smaller peak pupil dilation, indicating reduced listening effort with the added HA signal processing [25].

Although there is some utility of pupillometry to assess the impacts of DNR on listening effort, evidence on the impacts of different variations and settings of DNR on listening effort is still limited. HA manufacturers provide the option for audiologists to choose the strength of the DNR feature while setting the HA for their patients. These DNR strength settings typically range from DNR off to DNR maximum with several steps in between. To the best of our knowledge, no prior research studies have used pupillometry alongside self-report measures to examine the impacts of different DNR strengths on listening effort. This information will add to the evidence base for audiologists when recommending DNR strengths with the aim of reducing listening effort. Thus, the current study aims to identify the impacts of the strength of DNR algorithms in digital HAs on individuals’ listening effort. We measured subjective and objective listening effort in young adults with normal hearing during a speech-in-noise perception task and assessed the impacts of changes in DNR strength. Based on existing literature, we hypothesized that, as the DNR strength increased, participants’ listening effort would reduce when assessed using both self-report and pupillometry.

## 2. Materials and Methods

### 2.1. Participants

Eleven normal hearing young adults (5 females; M = 24, SD = 1.7 years) participated in this pilot study. The sample size was comparable to previous studies examining pupillometry and CRM speech perception using both between and within-subject designs (10–15 listeners) [26,27,28]. Normal hearing sensitivity was defined as audiometric thresholds ≤ 20 dB HL for octave frequencies from 250 to 8000 Hz. Normal hearing individuals were chosen based on previous pupillometry studies using simulated stimuli [23,24]. Given the exploratory nature of our study, the use of normal hearing young adults avoided the confounding effects of hearing loss (e.g., loss of spectral resolution, distorted auditory signals) that may have impacted the results. Similar DNR algorithms have also been used by normal hearing individuals when listening in challenging environments. Many wearable and hearable devices other than HAs also employ similar DNR algorithms to improve listening effort [8,9,10]. However, we note that using normal hearing individuals limits the generalizability of the results of the DNR technology on listening effort to listeners with normal hearing only. We aimed to understand how these parameters affect perception first in listeners without hearing loss. Participants were recruited from the University of Memphis student body. Individuals with no psychological or neurological problems, normal or corrected vision, and English as their primary language were included in this study. Participants were compensated monetarily for their time and gave written informed consent in accordance with procedures approved by the University of Memphis Institutional Review Board (IRB # 2370).

### 2.2. Stimuli

We used sentences from the Coordinate Response Measure (CRM) corpus [29]. These sentences consist of a call sign followed by a carrier phrase containing a target color–number combination (e.g., “Ready Charlie, go to *blue one* now”) spoken by 4 female and 4 male talkers. There were four possible colors (Blue, Red, White Green) paired with eight possible numbers (1–8), resulting in 32 response combinations. We used 64 sentences from the corpus recorded by one of the female talkers (female talker 3). Sentences were degraded by mixing the stimuli with a novel 6-talker CRM babble at 0 dB signal-to-noise ratio (SNR). A 0 dB SNR was selected as it is a level where perception begins to rapidly deteriorate for CRM sentences [28]. This is considered a less favorable and challenging listening condition for CRM stimuli and many other types of stimuli used to assess listening effort [30,31]. The noise babble was created in Audacity using a mixture of CRM sentences from 3 female talkers (that were not the target talker) and 3 male talkers. The same sentences from the 6 talkers were used to create the babble. Using CRM sentences as the competing babble has similar masking effects to other speech babbles [32].

### 2.3. Hearing Aids with Digital Noise Reduction

A premium digital receiver-in-the-canal wireless HA was used to evaluate the effects of DNR on listening effort. This HA processed data in 64 channels, more than 100 times per second, and used their 360° soundscape in all listening situations. This HA used a fast-acting DNR [33] achieved via a multi-microphone spatial-based noise reduction. An adaptive beamformer creates a back-facing cardioid response that serves as the noise estimator. In the fitting software, there are four DNR settings to select. These are DNR off, DNR minimum, DNR medium, and DNR maximum. The noise reduction increased by 3 more dB every time the strength was increased from one level to the higher one. We ensured that the HA’s output and DNR function were verified in an electroacoustic test box, per ANSI standards [34], before using it for this project. The HA was programmed using occluded coupling for a flat moderately severe hearing loss and the directionality feature was switched off to isolate the effects of the DNR system. An occluded fitting was used for stimulus generation as it optimally reflects the impacts of the DNR algorithm across the frequency range [35].

### 2.4. Speech Processing

The CRM sentences were mixed with the babble maskers and presented to the HA in each of the DNR conditions. A stereo sound file was created to have a target speech stimulus in one channel and noise in the other. Both the sentences and masker noise were presented with simultaneous onset and the presentation was controlled by a GSI 61 clinical audiometer. HA-processed stimuli were recorded using a KEMAR acoustic manikin, equipped with IEC 60,711 Ear Simulators. This manikin was placed at the center of a 10 × 10 ft^2^ double-walled sound-treated room. Based on the capabilities of the available KEMAR, processed stimuli were recorded only from the right HA connected to the right ear of the KEMAR. Two loudspeakers (front and behind) were placed at 1-meter distance relative to the KEMAR. Target sentences were presented from the loudspeaker at 0° azimuth (in front) relative to the HA, whereas babble was delivered at 180° azimuth (behind). Both the sentences and noise were presented at 65 dB SPL (0 dB SNR). Presentation level of 65 dB SPL was selected to represent typical conversational speech. Noise was presented at 0 dB SNR to trigger DNR activation. Output of the KEMAR was processed using a real-time analyzer.

### 2.5. Task

Participants were seated in the center of a 10 × 10 ft^2^ double-walled sound-treated room. The eye tracker (Gazepoint GP3 eye tracker, Gazepoint Inc., Vancouver BC V6H 1C3, Canada) and a computer monitor were located in front of the participant at a distance of ~25 inches. Stimuli were presented via circumaural headphones (Sennheiser HD 280, Sennheiser, Old lyme, CT, 06371, Canada). Target processed stimuli were randomized within and between the DNR conditions and also counterbalanced across participants according to a Latin square sequence to control possible learning effects [36]. The task was presented via a custom program coded in MATLAB 2019b (The MathWorks, Inc., Natick, MA, 01760, USA).

On each trial, participants heard one sentence; only a cross hair (+) appeared on the screen during stimulus presentation. Following, they were asked to identify the target color-number combination as quickly and accurately as possible. They were instructed to focus on the target sentences that stated the call sign of “Charlie”. They logged their response using a mouse and a 4 × 8 visual grid that appeared on the screen after the sentence presentation. The grid contained all 32 color-number combinations. Speech recognition scores were calculated for each DNR as the percentage of color-number combinations correctly recalled. Chance performance was 3.13% (=1/32). We required recall of both items for a correct response. Response reaction times (RTs) were also logged, measured as the time interval between stimulus presentation and the listener’s response. Mean RTs were calculated per listener and DNR condition and used for analyses.

### 2.6. Self-Report Measures

Self-reported listening effort was also assessed periodically during the CRM speech task. Listeners were asked to rate how effortful it was to perform the behavioral task using a seven-point scale [13]. The Likert-type scale ranged from no effort (1) to extreme effort (7) and was a continuous scale. The participant used a slider to indicate their effort. Ratings were probed every 20th stimulus and were subsequently averaged to measure self-reported listening effort for each DNR condition.

### 2.7. Pupillometry

Listeners’ gaze fixations were acquired using a Gazepoint GP3 eye tracker. This device uses infrared signal through a camera mounted on a desktop in front of the participant to measure eye gaze within ~1° visual angle accuracy. Validation studies have shown the GP3 desktop eye tracker achieve 92.5% sensitivity and 76.8% specificity with regard to positional eye gaze, which is comparable to other research-grade devices [30]. The Gazepoint device was positioned on a small desktop tripod just in front of the 24” computer screen. During the entire duration of testing, the lights in the testing room remained off to avoid evoking pupillary reflex dilations. If needed, participants were allowed to use contact lenses. Eye tracking data were continuously recorded from both eyes every 16.6 ms (i.e., 60 Hz sampling rate). A MATLAB API program was used to log the output of the eye tracker. Before every set of stimuli presentation and eye tracking recording, the tracker was re-calibrated. Calibration was performed using the tracker’s internal calibration routine with both eyes calibrated at 9-points across the horizontal/vertical dimensions of the screen. This calibration allowed us to ensure that the individual’s eyes were in alignment with the computer screen. To promote comfort, no chin nor head rest was used during the task. Accuracy of eye gaze position on the screen is between a 0.5° and 1° visual angle. Calibration error of the GP3 is also similar with or without chin rest support [37]. Validation studies further show that while the absolute size of peak pupil dilation (PPD) is slightly smaller (1.4 mm) when measured using the GP3 vs. other research-grade eye trackers with chin rest, the relative changes in PPD with speech SNR are comparable between devices. For instance, in their study on pupillometry and listening effort of noise-degrade speech, Rahme et al. [38] concluded “…the pattern of pupillary changes recorded with the Gazepoint GP3 HD was similar to that recorded using a gold standard pupillometry device [EyeLink 1000], with moderate between-device agreement and no interaction between device and noise level on pupil dilation responses.” Participants were instructed to remain still in the upright recliner chair following calibration. Compliance was monitored by the experimenter and within the data as dropped trials (see below).

Pupil responses were continuously recorded during the speech recognition task. The onset of each stimulus presentation was time-stamped in the data file which allowed us to analyze time-locked changes in eye data for each stimulus. A “+” symbol appeared on the center of the computer screen throughout the speech recognition task to maintain a central fixation gaze for the participants and ensure constant luminance on the screen. (The color-number response screen appeared after sentences were competed.) Recordings were then filtered through a passband of 0.001–15 Hz, epoched [−500–5000 ms] (where *t* = 0 marks sentence onset; *t* ≈ 3000, sentence offset), and baselines were corrected to the pre-stimulus interval [−500–0 ms]. Pupil responses were then averaged separately for each DNR condition. The eye tracker also logged every time the participant blinked during the task. Any data segments contaminated with these artifacts were linearly interpolated prior to analysis. Per the Gazepoint documentation (https://www.gazept.com/downloads/?v=0b3b97fa6688 (accessed on 9 September 2025)), the GP3 device monitors pupil center corneal reflection (PCCR). Blinks are automatically detected by monitoring the loss of PCCR signal when the eyelid closes. This identifies data gaps in the eye image captured by the camera during blinks and these events are automatically logged as a blink event in the recording. Across subjects and conditions, data loss (and interpolation) due to the events were limited to 22 ± 12% of data samples from the entire recording (i.e., raw pupil time courses). Furthermore, measures were taken to apply corrections for any subtle changes in the distance between the eye tracker camera and the participant that could affect pupil measurements (e.g., during head movement). To do this, the eye tracker recorded a continuous scale factor for each pupil, where a scale value of 1 represents pupil depth (distance to the camera) at the time of calibration, scale value of <1 represents that the user is closer to the eye tracker, and a scaling value of >1 represents that the user is further away. This scale factor was then used to weight the running time course prior to averaging and correct for movement artifacts. On average, <5% of epochs (4.65 ± 0.43 across subjects/conditions) were lost to artifacts and were discarded.

### 2.8. Statistical Analyses

Descriptive statistics were used to visualize data distributions. Pupil responses from both eyes were first collapsed and then analyzed in terms of their latency and amplitude characteristics. Visual inspection of the grand averaged waveforms revealed that pupil responses began to increase dramatically ~1 s before stimulus cessation and achieved their maximum (saturation) by 1–2 s post-stimulus as listeners executed their behavioral response. We quantified this pupil response latency by measuring the relative risetime for waveforms to transition between 20 and 80% of their maximal amplitude using the *risetime()* function in MATLAB. We reasoned faster latencies would be indicative of more efficient speech processing and therefore reduced listening effort [26]. A mixed-model ANOVA was performed to assess differences across DNR conditions for behavioral (percent accuracy and reaction time), self-report (listening effort), and pupillometry (amplitude and latency) variables. Subjects were included as a random factor. The α-level for significance was *p* = 0.05. These analyses were performed using SAS 9.4 (SAS Institute, Inc. San Francisco, CA, 94108, USA).

Furthermore, we performed repeated measures correlation (rmcorr) analyses to assess the association between perceived listening effort ratings and pupillometry amplitude as well as latency [39]. This analysis, as opposed to Pearson’s or Spearman’s correlation, was chosen to account for within-subject associations and achieve a relatively higher degree of statistical power with a limited sample size. All rmcorr analyses and plots were completed using R Studio version 1.4.1717.

## 3. Results

### 3.1. Behavioral and Self-Report Data

**Figure 1:**
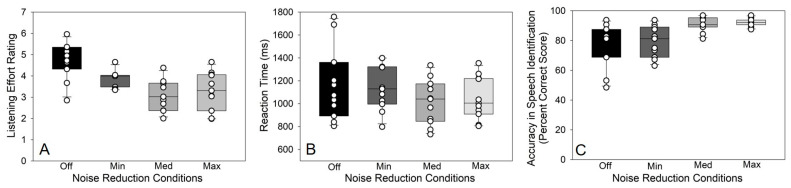
Strength of hearing aid dynamic noise reduction (DNR) impacts listening effort, accuracy of speech identification, and reaction time. (**A**) Perceived listening effort (self-reports) with increasing strength of DNR applied to noise-degraded speech stimuli; (**B**) reaction times with changes in DNR; and (**C**) Speech identification accuracy (percent correct score). Error bars = ± 1 s.e.m. Off, Min (Minimum), Med (Medium), and Max (Maximum) noise reduction. Circles on each bar represent individual data points for each participant.

Figure 1A shows self-reported listening effort for participants across the four DNR conditions. Self-reported listening effort declined with increasing strength of DNR [*F_3,30_* = 14.49, *p* < 0.0001]. Tukey-adjusted post hoc pairwise comparisons revealed reductions in effort when DNR was off through medium DNR, with effort plateauing thereafter (DNR off to minimum: *t_30_* = 2.92, *p* = 0.03; DNR off to medium: *t_30_* = 5.97, *p* < 0.001; DNR off to maximum: *t_30_* = 5.25, *p* < 0.001; DNR minimum to medium *t_30_* = 3.05, *p* = 0.02). This suggests that DNR effectively reduced perceived listening effort until a medium level setting, at which point the effect saturated with further increases in DNR strength. No other significant differences were found (DNR minimum to maximum *t_30_* = 2.33, *p* = 0.11; DNR medium to maximum *t_30_* = −0.72, *p* = 0.88).

Figure 1B shows the average RTs across the four DNR conditions. Though medium DNR appeared to yield the fastest responses, an ANOVA revealed that RTs were invariant to changes in DNR [*F_3,30_* = 1.50, *p* = 0.23]. Thus, DNR had no impact on the speed of listeners’ speech identification. Figure 1C shows accuracy of speech identification (percent correct score) across the four DNR conditions. There was a notable improvement in speech recognition accuracy with changes in DNR [*F_3,30_* = 9.41, *p* = 0.0002]. Post hoc pairwise comparisons revealed improved accuracy of speech identification with DNR medium compared to DNR off (*t_30_* = −3.90, *p* = 0.003), DNR maximum compared to DNR off (*t_30_* = −4.21, *p* = 0.001), DNR medium compared to DNR minimum (*t_30_* = −3.23, *p* = 0.015), and DNR maximum compared to DNR minimum (*t_30_* = −3.54, *p* = 0.007). These findings suggest that listeners required at least a medium strength of DNR to show improvement in speech identification accuracy. No other significant differences were found (DNR off to minimum *t_30_* = −0.67, *p* = 0.90; DNR medium to maximum *t_30_* = −1.14, *p* = 0.99). There were no other correlations between any of the behavioral and self-reported variables.

### 3.2. Pupillometry Data

**Figure 2:**
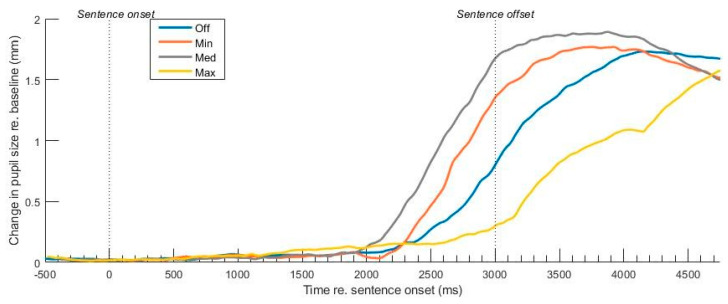
Stimulus-evoked pupil responses as a function of DNR strength. *t* = 0 reflects the onset of sentence. Pupil dilation peaks near the offset of speech as listeners prepare to report the target letter–number combination of CRM sentences.

Figure 2 displays the grand averaged waveforms of the pupil dilation amplitude and latency characteristics, indicating DNR-related modulation in pupil waveforms between 2000–4000 ms post-stimulus onset. In this time window, the sentence offset is at around 3000ms. We measured pupil response amplitudes just before the stimulus offset, at 2700 ms. This timepoint preceded both the stimulus cessation and listeners’ behavioral response. Changes in pupil size from baseline increased as DNR strength increased from off to moderate DNR but then reduced notably for maximum DNR.

**Figure 3:**
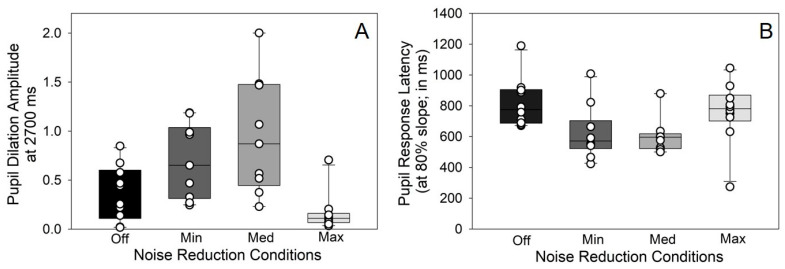
Pupil responses during speech perception are modulated by strength of DNR. (**A**) Differences in the average pupil dilation (i.e., amplitude at 2700 ms) as the strength of DNR changes; (**B**) pupil response latency (i.e., waveform slope risetime). Error bars = ± 1 s.e.m. Circles on each bar represent individual data points for each participant.

Figure 3A shows the average pupil response amplitudes (at 2700 ms latency) across the four DNR conditions. Pupil response amplitude was strongly modulated by the strength of DNR applied to the speech stimulus [*F_3,26_* = 13.66, *p* < 0.0001]. Post hoc comparisons revealed differences in pupil amplitude between DNR off and DNR medium (*t_26_* = −4.32, *p* = 0.0011), DNR minimum and DNR maximum (*t_26_* = 4.17, *p* = 0.0016), and DNR medium and DNR maximum (*t_26_* = 5.86, *p* < 0.0001). This indicates that pupil dilation increases with the strength of the DNR until medium strength and then as DNR reaches maximum strength, pupil dilation reduces.

The latency of pupil responses was quantified by measuring the relative risetime for waveforms to transition between 20 and 80% of their maximal amplitude (Figure 3B). Pupil response latency varied with DNR [*F_3,26_* = 5.15, *p* = 0.0063], but again in a non-monotonic way. Post hoc pairwise comparisons revealed that pupil latency significantly reduced as the DNR strength increased from off to minimum (*t_26_* = 3.15, *p* = 0.0198) and off to medium (*t_26_* = 3.20, *p* = 0.0178). No other significant differences were observed.

### 3.3. Agreement Between Self-Report and Pupillometry Data

**Figure 4:**
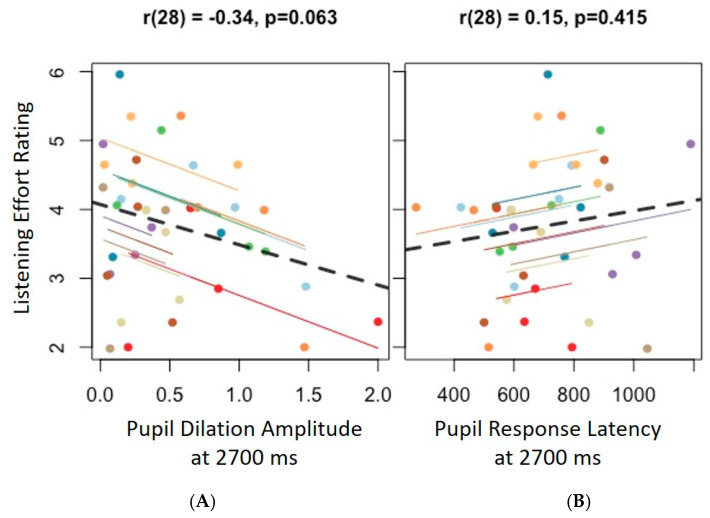
Rmcorr scatter plots demonstrating within-subject associations between perceived listening effort ratings and pupil response characteristics. (**A**) Perceived listening effort ratings vs. pupil dilation amplitudes; (**B**) perceived listening effort ratings vs. pupil response latency. Observations from each participant are plotted using one of the colors, along with their corresponding rmcorr regression slope (dotted = n.s.). A single dot for a participant represents the values for each of the tested DNR conditions. Listening effort ratings represent perceptual effort after each block of stimuli and pupil responses were plotted at 2700 ms.

To assess the reliability between self-report and pupillometry measures of listening effort, repeated measures correlation analyses (rmcorr) were performed. Data across the DNR conditions were aggregated for this analysis, allowing us to assess potential relations between pupil and behavioral data across the four DNRs within each listener. Figure 4A,B show the rmcorr scatter plots, demonstrating the correlations between perceived listening effort ratings and amplitude (4A) and latency (4B) of pupil responses. Visual inspection of scatter plots did not show any notable correspondence between the two measures. Rmcorr analyses between listening effort rating and pupil response amplitude demonstrated a marginally negative correlation (*r* = −0.34, *p* = 0.06). This suggests that there is trend where pupil response amplitude reduces as participants’ perceived listening effort increases. No notable or significant correlations were found between perceived listening effort and pupil response latency.

## 4. Discussion

Other than improving speech understanding (in noise), reducing listening effort is one of the primary goals audiologists aim to achieve after HA fitting. As task demands increase, making it difficult to understand conversation in adverse listening situations, DNR in HAs is often activated with the goal of reducing unwanted background noise and listening effort. This study assessed normal hearing individuals’ listening effort while listening to speech-in-noise stimuli processed through HAs set at different DNR strengths using self-report measures and pupillometry. Overall, our results suggest changes in subjective and objective assays of listening effort as the DNR strength changes, though little relation between self-report and pupillometry measures.

### 4.1. Effects of DNR on Self-Report Listening Effort

When DNR was off, our participants reported maximum effort. As the DNR strength increased from off to medium, listening effort reduced. This suggests that participants could understand the speech better in the presence of the babble noise and thus they perceived less effort. However, further increase in DNR strength to maximum did not yield further improvements in listening effort, i.e., listeners’ perceived effort tended to saturate. Overall, our participants reported least listening effort in the medium DNR condition. This might be due to optimum release from masking at this strength. However, as the DNR strength reached maximum, there was a slight increase in effort. This can be attributed to possible distortion introduced by aggressive DNR processing [40]. Slight changes in RT supports the previous literature [14]. However, lack of statistical significance might be attributed to possible contamination of any recall component that participants used before responding [18].

### 4.2. Effects of DNR on Pupil Dilation as a Measure of Listening Effort

Conventionally, research has suggested that when task demands increase, such as in more difficult listening situations, pupil dilation amplitude and peak latency systematically increase [22]. Overall, our results revealed a main effect of strength of DNR on pupil dilation, which aligns with previous studies using pupillometry [25]. Winn et al. [41] on their normal hearing participants also reported similar pupil responses in a speech task. Additionally, they indicated that the responses are not time-locked to the stimulus and there is lack of reliability in latency differences potentially due to task-evoked boredom.

In contrast to the pupil latency response, we found that pupil dilation amplitude did not align with other studies [42]. However, Fielder et al. used individuals with hearing loss and continuous speech stimuli as opposed to normal hearing participants and sentence stimuli in our study. For our participants, pupil dilation amplitude was lower in more difficult DNR conditions such as DNR off. Participants had the maximum pupil amplitude in DNR medium, which was perceived as the easiest condition. Wang et al. [43] reported similar responses with smaller peak task-evoked pupil responses with increase in daily fatigue. Stronks et al. [31] used both normal hearing and cochlear implant individuals and the pupil responses were different in both groups. These discrepancies across studies may suggest that other stimulus- and listener-related factors like syntactic complexity and motivation might contribute to changes in pupillometry results [3,44].

Alternatively, increased pupillary responses could be a marker of higher speech intelligibility rather than listening effort, per se. For example, if more words are heard in a condition, more linguistic units need to be processed per unit time, which could increase processing load and increase pupil dilation. However, we find this explanation unlikely given that speech recognition and pupil responses showed different patterns across DNRs, with behavior increasing monotonically and pupil responses nonmonotonically, respectively.

Additionally, the use of normal hearing individuals may have changed the way participants performed. For example, Winn et al. [41] suggested that normal hearing individuals might find these listening situations less demanding or could get bored and this could result in unreliable pupil responses. High performance in all the speech perception testing across conditions might have also led to less engagement, leading to our contradictory pupil amplitudes compared to those in the literature. As the situation was easy, this might have reduced their motivation to respond leading to reduced pupil size. Future research should consider replicating the present results on a larger sample and in individuals with hearing loss. As this was the first study to assess the impacts of the strength of the DNR algorithm, replicating the study on a larger population will improve generalizability. Additionally, future research should better titrate task difficulty to maximize chances of observing differences in listening effort.

### 4.3. Agreement Between Self-Report and Pupillometry Findings

Consistent with previous literature [17], our results demonstrated a lack of correlation between self-report and pupillometry measures of listening effort. Lack of agreement suggests that the two measures are evaluating different underlying aspects of listening effort and/or that listening effort is not driven by a single phenomenon [17,18]. Indeed, subjective vs. objective measures of listening effort may not be entirely isomorphic and reflect different underlying mechanisms of attention, arousal, and awareness [13]. Other than listening effort, pupil responses have been used to index cognitive processing load—as indicated in the review by van der Wel and van Steenbergen [45]—tiredness [46], and arousal levels [43]. Extensive research has demonstrated that tasks requiring higher cognitive demands often result in an increase in pupil dilation [45]. This overlapping involvement of cognitive processes with pupil responses reflect that pupil dilation changes in our participants might not be purely because of the auditory listening task demands. McGarrigle et al. [46] in their study comparing self-report and pupillometry measures reported significant negative association between task-evoked pupil responses and tiredness but not perceived effort. As their participants reported increased tiredness, there was a reduction in pupil responses, suggesting a possibility of resource depletion in the presence of tiring listening situations. This indicates that pupil responses might be an indicator of physiological arousal. The changes in pupil response amplitude in our participants as the DNR condition changed might be attributed to changes in tiredness and not listening effort. Another possible reason for lack of correlation could be due to inherent differences in the way both measures are completed. Self-report measures are usually completed as a single data point after a set of stimuli, whereas pupillometry records point-to-point responses across each stimulus. It is possible that participants experienced some level of recall bias and could not accurately reflect their perceived effort [47].

In reviewing the Framework of Understanding Effortful Listening (FUEL) [3], Keur-Huizinga et al. [48] pointed out that multiple underlying mechanisms such as task performance, working memory, and processing are responsible for listening effort and different measures are sensitive to different processes. Thus, a more multimodal approach may be needed for listening effort measurement that includes tests in different modalities.

While our findings provide useful insights, certain limitations must be taken into account. First, we acknowledge the smaller sample size. Although there are several large sample research studies indicating the impacts of listening effort on pupillometry results, and also the impacts of DNR, this study for the first time looked at changes in the strength of the DNR algorithm. Future studies should replicate the current methods on a larger population to assess the robustness and generalizability of our findings at different DNR strength settings for different hearing aid manufacturers. Second, use of normal hearing individuals may not have been ideal to reflect the consequences of hearing loss that may impact listening effort. However, similar DNR algorithms have been used by normal hearing individuals and researchers have reported improvements in listening effort [8,9,10]. Care should be taken while interpreting these results for individuals with hearing loss. Future research should test individuals with hearing loss, with and without hearing aids, and with different DNR strengths to clarify the current study results. Finally, although recent research shows that the GP3 eye tracker used in this study without a chin rest has comparable results to that of other research-grade eye trackers with chin rests, not having a chin rest might have introduced errors. Care should be taken while interpreting the results and translating it to other eye tracker results.

## 5. Conclusions

Listening effort is a multifaceted phenomenon that is influenced by several stimulus-related and listener-related factors, and improvements in listening effort using DNR in HAs depends on these factors. Assessment of listening effort through different measurement methods can reflect different constructs and care should be taken while correlating results of different methods. Our study demonstrated that changes in DNR strength could successfully be reflected in changes in perceived listening effort. This would provide clinical audiologists with information on customizing the DNR strength settings (whether for HAs or other hearing wearables in listeners with/without hearing loss) based on their listening comfort requirements. Additionally, pupillometry can provide information on differences in listening effort at different DNR strengths. By objectively tracking pupil data under different noise conditions, clinicians can determine whether DNR settings could effectively reduce cognitive load or effort without compromising speech understanding. These measurements can help guide individualized rehabilitation strategies by indicating necessary fine tunings or targeting additional listening communication strategies in difficult listening situations. The disagreement across the measures indicates that more research is required to provide more conclusive information on the aspects of effort each measure taps and understand the relation (or lack thereof) between pupil responses and auditory perceptual outcomes. Our results, along with the existing literature, suggest that there is probably no gold standard measure to unequivocally capture listening effort. Selection of different measures should depend on the specific underlying process one intends to evaluate. Future researchers should carefully consider methodological choices as well as the different factors influencing listening effort while choosing the methods to be used to assess the impacts of HA features.

## Data Availability

The datasets generated during and/or analyzed during the current study are not publicly available due to lab policies but are available from the corresponding author on reasonable request.

## Abbreviations

The following abbreviations are used in this manuscript:

HA	Hearing Aid
DNR	Digital Noise Reduction
CRM	Coordinate Response Measure
ANSI	American National Standard Institute
KEMAR	Knowles Electronic Manikin for Acoustic Research
MATLAB	Matrix Laboratory
FUEL	Framework of Understanding Effortful Listening
